# DNA alterations in Cd133+ and Cd133- tumour cells enriched from intra-operative human colon tumour biopsies

**DOI:** 10.1186/s12885-017-3206-8

**Published:** 2017-03-27

**Authors:** Diana Cervantes-Madrid, Yvonne Wettergren, Peter Falk, Kent Lundholm, Annika G. Asting

**Affiliations:** 1 Department of Surgery, Institute of Clinical Sciences, Sahlgrenska Academy, University of Gothenburg, Gothenburg, Sweden; 2 Department of Surgery, Sahlgrenska University Hospital, S-413 45 Gothenburg, Sweden

**Keywords:** Cancer stem cells, CD133/Prominin1, colorectal cancer, copy number variations, DNA alterations

## Abstract

**Background:**

Tumour stem cells are considered important to promote disease progression, recurrence and treatment resistance following chemotherapy in colon cancer. However, genomic analyses of colorectal cancer have mainly been performed on integrated tumour tissue consisting of several different cell types in addition to differentiated tumour cells. The purpose of the present study was to compare genomic alterations in two cell fractions enriched of CD133+ and CD133−/EpCAM+ cells, respectively, obtained from fresh intraoperative human tumour biopsies.

**Methods:**

The tumour biopsies were fractionated into CD133+ and CD133−/EpCAM+ cells by immunomagnetic separation, confirmed by immunocytochemistry and Q-PCR. DNA were extracted and used for array comparative genome hybridization (aCGH) after whole genome amplification. Frozen tumour tissue biopsies were used for DNA/RNA extraction and Q-PCR analyses to check for DNA alterations detected in the cell fractions.

**Results:**

The number and size of DNA alterations were equally distributed across the cell fractions; however, large deletions were detected on chromosome 1, 7 and 19 in CD133−/EpCAM+ cells. Deletions were frequent in both cell fractions and a deletion on chromosome 19p was confirmed in 90% of the patients.

**Conclusion:**

Isolation of enriched cells derived from tumour tissue revealed mainly genomic deletions, which were not observed in tumour tissue DNA analyses. CD133+ cells were genetically heterogeneous among patients without any defined profile compared to CD133−/EpCAM+ cells.

**Electronic supplementary material:**

The online version of this article (doi:10.1186/s12885-017-3206-8) contains supplementary material, which is available to authorized users.

## Background

Cancer stem cells (CSC) have been related to various properties of aggressive tumours like metastasis, chemo- and radio-resistance, relapse, and poor prognosis [1]. A CSC is regarded a cell within tumours able of self-renewal and production of heterogeneous lineages of tumour cells that comprise solid tumours. Current chemotherapies that target proliferating cells are assumed to meet considerable levels of resistance from CSC in solid tumours since CSC proliferate at slow rates compared to differentiated tumour cells and normal cells [2]. Studies have displayed that patients with colorectal cancers containing CD133 expressing cells related to reduced survival and high risk of early recurrence [3]. This fact is important, particularly in the light of that CD133 is regarded a relevant marker for cancer stem cells in colorectal tumours, although its cell functions are unknown and it may be that only a small fraction of CD133+ cells have stem/progenitor activity [4, 5].

Understanding genetics of cancer cells, including CSC, is important since the use of targeted therapy for cancer treatment may be increasingly important. Also, genomic imbalances or copy number variations (CNVs) correlate with gene expression levels – suggesting direct effects on gene expression by changes in gene copy numbers [6]. However, little is known about genomic changes of CD133+ cells compared to differentiated tumour cells within solid tumours. Therefore, the aim of the present study was to compare genomic changes in CD133+ cells versus differentiated (CD133−/EpCAM+) enriched tumour cells from intraoperative human tumour biopsies.

## Methods

### Patients

Twenty-seven patients diagnosed with colon cancer selected for curative surgery and who did not receive any chemotherapy treatment prior to biopsy collection, according to institutional guidelines at the time, were included in this study (2013–2014). Written, informed consent was obtained from all patients. The study protocol was approved by the Board of Ethics at the University of Gothenburg (permit number: 365–05). Patient characteristics, after exclusion of one patient whose tumour was later confirmed to be an adenoma, are shown in Table 1. Two tumour biopsies were collected from each tumour; one was immediately frozen in liquid nitrogen and the other placed in tissue storage solution for cell separation.

**Table 1:** Patient characteristics (*n* = 26)^a^

	Patients (n)
Male/Female	11/15
Age at surgery	71.1 (44–94)
Tumour stage (I-IV)	I 5, II 10, III 9, IV 2
Tumour differentiation	Medium 23/High 2/Mucinous 1
Positive lymph nodes (analysed 5–52)	1 (0–6)
Tuymour location	Right 14 / Left 11/Multiple 1
Radical surgery Y/U/N	23/2/1
Recurrent disease (June 2015)	3

^a^ One patient with adenoma excluded. Data presented as mean (range), Y = yes/U = uncertain/N = no

### MACS tumour sample separation

**Fig. 1:**
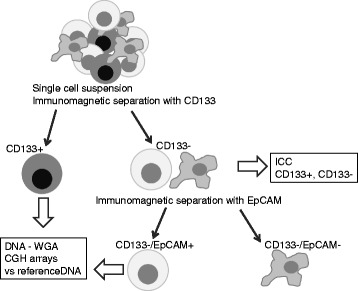
Tissue separation and cell fractionation Tumour tissue was separated into CD133+ and CD133−/EpCAM+ cell fractions confirmed with ICC and analysed with CGH microarrays

The biopsy designated for cell separation was kept in pre-chilled MACS Tissue Storage Solution and used within 24 h. The samples were dissociated into single cell suspensions using Tumour Dissociation Kit (MACS, Miltenyi Biotec, Bergisch Gladbach, Germany) by mechanical dissociation and enzymatic degradation of extracellular matrix. The single cells were incubated with Dead Cell Removal Microbeads for 15 min at room temperature and separated with MACS LS Column; the labelled cells were collected as the effluent fraction. Cells were washed with 9 ml stock solution (1.25 ml 20X Binding Buffer Stock Miltenyi Biotec diluted up to 25 ml with ddH_2_O) and centrifuged at 300G for 10 min and labelled with CD133 antibodies conjugated to ferromagnetic beads (CD133 Microbeads, 130–050-801, MACS Miltenyi Biotec) for 30 min at 4 °C. After incubation, cells were washed with 9 ml MACS Buffer, centrifuged at 300G for 10 min, separated using MACS LS column and kept on ice until further analyses (Fig. 1). CD133 negative cells were used in a second step for separation labelled with EpCAM antibodies (CD326, 130–061-101, MACS Miltenyi Biotec) for 30 min at 4 °C and washed with MACS Buffer before separation. Cells were passed through MACS LS column. The negative fraction was collected as the effluent (CD133−/EpCAM-) and positive cells were flushed out of the column and collected as EpCAM positive fraction (CD133−/EpCAM+). Cells were used immediately for downstream analysis after separation of CD133+, CD133−/EpCAM+ and CD133−/EpCAM- fractions.

There are some technical issues to consider with studies of genomic alterations in small amount of cells as performed in the present study. One such matter is that CD133+ cells are few within tumours. Therefore, whole genome amplification (WGA) was necessary before microarray analyses, which may affect amplification and interpretations of microarray results [7]. We chose to use a Multiple Displacement Amplification (MDA) technique that was recommended for CGH microarrays (Agilent) in detection of amplifications and deletions of DNA. Also, the EpCAM+/Cd133- cell fraction was collected by additional round of enrichment with column elusion, which may reduce recovery. However, unselective difference in recovery of cell fractions should not impact seriously on the possibility to perform qualitative comparisons. The DNA quality was checked along preparations on all samples.

### Immunocytochemistry for CD133 detection

Biopsies from four random patients were separated with CD133 antibody as described above (MACS, Miltenyi Biotec, Bergisch Gladbach, Germany) and the two fractions obtained, CD133+ and CD133-, were used for immunocytochemistry. The cells were attached to a glass slide using cytospin centrifugation at 1000 rpm for 4 min. The slides were placed in methanol at −20 °C for 20 min for fixation. After this step, the MACH2 staining protocol was followed (Histolab, Gothenburg, Sweden). Briefly, peroxide was blocked with peroxidised 1 reagent (Histolab, Gothenburg, Sweden) for 5 min. After washing with TBS wash buffer (Histolab, Gothenburg, Sweden), cells were covered with Background Sniper (Histolab, Gothenburg, Sweden) for 10 min to avoid non-specific binding. TBS wash buffer was used in all washing steps. Cells were incubated with CD133 primary antibody (AC133, 130–090-422, Miltenyi Biotec, 15 μg/ml) overnight at 4 °C, washed, and incubated with MACH2 30 min for secondary ALP detection. After washing, cells were covered with Warp red chromogen (prepared according to data sheet) for 7 min and counter stained with hematoxylin for 2 min. The slides were washed with tap water and mounted using aqueous mounting medium. Mouse IgG1 (Dako 0931, 15 μg/ml) was used as negative control.

### DNA and RNA isolation

DNA and RNA were isolated from CD133+ and CD133−/EpCAM+ cells according to Allprep DNA/RNA micro Kit (Qiagen, Hilden, Germany) and from tumour tissue with Allprep DNA/RNA mini kit (Qiagen, Hilden, Germany). Briefly, cells were lysed and homogenized with RLT Buffer and transferred to an Allprep DNA spin column for binding of genomic DNA. The flow through was mixed with 70% ethanol and transferred to an RNeasyMinElute spin column for total RNA binding. To elute the RNA, 12 μl of RNase-free water were used and DNA was eluted with 50 μl of Buffer EB pre-heated at 70 °C. RNA quality and quantity were measured using a Bioanalyzer 2100 (Agilent Technologies, Santa Clara, CA, USA). Only RNA with RIN value ≥6.5 was used in further analyses. RNA samples were stored at −80 °C and DNA at −20 °C.

### Gene expression of stem cells markers

Gene expression levels of genes related with stemness were measured by Q-PCR in the CD133+ and CD133−/EpCAM+ cell fractions from randomly selected patients. Genes tested were *BMP7* (CD133+ *n* = 16, CD133−/EpCAM+ *n* = 17), *PROM1* (gene for CD133; CD133+ *n* = 10, CD133−/EpCAM+ *n* = 12), and *POU5F1/Oct4* (CD133+ *n* = 12, CD133−/EpCAM + =13). All three genes were not tested in all samples due to small sample size, poor RNA quality and/or low amount of RNA. *GAPDH* was used as a housekeeping gene, confirmed separately [8], and run for all samples. cDNA synthesis was performed using QuantiTect Reverse Transcription kit (Qiagen, Hilden, Germany). Q-PCRs were run in LightCycler 1.5 using LightCycler FastStart DNA Master plus SYBR Green I kit (Roche Diagnostics, Basel, Switzerland) with final primer concentration 0.5 mM for each gene. Primer information is described by Lönnroth et al. [9]. For each Q-PCR, 2 μl cDNA were used with the following PCR conditions: Activation for 10 min at 95 °C and denaturation for 10 s at 95 °C, 20 °C/s were the same for all reactions. Annealing: 7 s 58 °C (*PROM1*); 4 s 64 °C (*BMP7*, *GAPDH*); 5 s 66 °C (*OCT4B1*). Extension and cycle numbers: 22 s 72 °C, 40 cycles (*PROM1*); 5 s 72 °C, 45 cycles (*BMP7*); 20 s 72 °C, 40 cycles (*OCT4B1*); 5 s 72 °C, 40 cycles (*GAPDH*). PCR efficiency and slope of standard curve for *GAPDH* was 88.97% and −3.62, *BMP7* 83.44% and −3.79, *PROM1* 76.42% and −3.77, and *OCT4B1* 91.01% and −3.60. Q-PCR results were calculated according to the relative standard curve method and all samples were in the range of the standard curve. Negative controls were negative. Results were analysed with ANOVA followed by Fisher PLSD and are presented as mean units/units of GAPDH ± SEM. *P* ≤ 0.05 was considered statistically significant in two-tailed tests.

### DNA Whole Genome Amplification

DNA from CD133+ and CD133−/EpCAM+ populations as well as reference DNA (Agilent Euro male, #5190–3796) for CGH array analysis were amplified using the REPLI-g Single Cell Kit (Qiagen, Hilden, Germany) according to manufacturer’s protocol. Briefly, 2.5 μl of template DNA was incubated with 2.5 μl buffer D1 for 3 min. Neutralization buffer, N1, was added and REPLI-g master mix added after neutralization. The mixture was incubated for 2 h at 30 °C. DNA polymerase was inactivated at 65 °C for 3 min. Reference DNA was amplified in 8 aliquots that were pooled and used as reference DNA in the array CGH. DNA was stored at −20 °C.

### DNA Purification

DNA was purified using GFX PCR DNA and Gel Band Purification kit (Illustra, GE Healthcare, Little Chalfont, UK) according to manufacturer’s instructions. DNA samples were mixed with 500 μl of Capture Buffer type 3, loaded onto a GFX MicroSpin column and centrifuged at 16000G for 1 min. The column was washed with 500 μl Wash buffer type 1 and centrifuged (16000G, 1 min); this washing step was repeated to achieve high purity. DNA was eluted with 30 μl Elution buffer type 6 after 1 min incubation. DNA quality was checked on a 2% agarose gel and additional tests were run using 2200 Tape station (Agilent Technologies, Santa Clara, USA). DNA quantity was measured using Qubit® assay (Thermo Fischer Scientific, Waltham, MA, USA). Two patients were excluded from further analysis due to poor DNA quality. DNA was stored at −20 °C.

### Array CGH – DNA alterations in CD133+ and CD133−/EpCAM+ cell populations

Cell fractions of CD133+ and CD133−/EpCAM+ from 20 patients were used for study of differences in DNA alterations between the two cell populations (*n* = 40 arrays). 500 ng of WGA DNA isolated from CD133+ and CD133−/EpCAM+ cells were used to perform array CGHs against WGA reference DNA (Agilent Euro male, #5190–3796). Genome wide analyses of DNA copy number changes were performed using Sureprint G3 Human CGH Microarray Kit (Agilent Technologies, Santa Clara, CA, USA) format 4x180K with 13 kbp overall median probe spacing (11 kbp in Refseq genes) according to Agilent Oligonucleotide Array-Based CGH for Genomic DNA Analysis Enzymatic Labelling for Blood, Cells or Tissues Protocol version 7.1, December 2011. Slides were scanned with Agilent Microarray Scanner G2505C, fluorescence intensities were extracted using the Feature Extraction software program FE v10.7.1.1 (Agilent Technologies, Santa Clara, USA) and analysed using CGH Analytics software Genomic Workbench version 7.0.4.0 (Agilent Technologies, Santa Clara, USA). Aberration algorithm ADM-2 (threshold 6, fuzzy zero on, normalized with diploid peak centralization and GC correction) was used with default design level filter (v2) and feature level filter. Filter after analysis was DefaultAberrationFilter_v2 (minProbe 3, minAbs. average log ratio 0.25). Combined analysis of arrays divided into CD133+ and CD133−/EpCAM+ groups were performed with inter array analysis.

### Q-PCR – DNA alterations in tumour tissue

Q-PCR assays were performed at 4 regions of chromosome 19p to study if the deletions at chromosome 19p detected in cell fractions also were detected in corresponding tumour tissue. Well-known colon cancer alterations were also evaluated with Q-PCR; 1 region at chromosome 13 and 1 region at chromosome 20 as well as 1 region at chromosome 10 that is known to be unaffected in colorectal cancer (control) in DNA from tumour tissue biopsies. Primers were designed by TATAA Biocenter AB (Gothenburg, Sweden) with PrimerBlast (http://www.ncbi.nlm.nih.gov/tools/primer-blast/), primer sequences for chromosome 10, 13 and 20 are described elsewhere [10] and for chromosome 19 in Additional file 1: Table S1. The Q-PCR assays were validated on gBlocks to estimate the PCR efficiency of the assay. A seven point standard curve was generated with four replicates in each point and run in ten-fold dilution steps. The dilution series covered a template concentration between 2 × 10^7^ and 20 copies/reactions. LPA carriers were added to the dilution series to avoid unspecific interactions of target. Q-PCR analysis was performed with TATAA SYBR® GrandMaster® mix (TATAA Biocenter AB, #TA01) in 10 μl reactions on CFX384 instrument (Bio-Rad, Hercules, CA, USA). Two replicates of the DNA control (Agilent Euro male, #5190–3796) with concentration 20 ng/reaction were measured together with the standards. Raw Q-PCR data was analysed with GenEx software (version 6, MultiD Analyses AB), standard curve and limit of quantification were generated. Specificity control (amplicon size) for PCR products from designed assays was performed on capillary electrophoresis instrument (Fragment Analyser, Advanced Analytical Technologies, Ankeny, IA, USA) according to manufacturer’s instructions. Results were analysed according to comparative Cq method with the region at chromosome 10 and reference DNA (Agilent Euro male, #5190–3796) as standards. Results between 0.90–1.10 were regarded as not altered.

## Results

### Immuno-cytochemical detection of CD133 expression in the tumour cell fractions

**Fig. 2:**
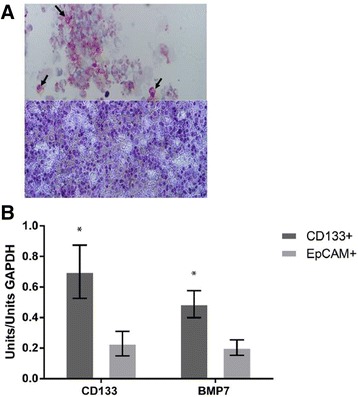
CD133+ and CD133- are various cell fractions. **a** Immuno-cytochemical staining of CD133 positive cells (top, black arrows added in the photo indicate examples of CD133+ cells) and CD133 negative cells (bottom), with a difference at 63% versus <2% overall staining among cells in the two fractions, which displays differences in CD133 expression in the two cell populations. **b** Transcript levels of stem cell markers in CD133+ and CD133−/EpCAM+ cell populations differed significantly (CD133/*PROM1 p* = 0.018, *BMP7 p* = 0.0081)

CD133 protein expression was evaluated in 4 samples by immunocytochemistry to demonstrate that CD133+ and CD133- cell fractions were separated into two cell populations with different expression of CD133. The results display CD133 protein expression in 63% (range 51–74%) of enriched cells in the CD133+ fraction and <2% (range 0–3%) of CD133- cells (Fig. 2a).

### Gene expression of stem cell markers

Transcript levels of known cancer stem cell genes indicated that the CD133+ cell population expressed higher levels of such genes. *PROM1* (*PROM1* = CD133 gene) displayed increased expression compared to cells in the CD133−/EpCAM+ population (*p* = 0.018) (Fig. 2b). CD133+ cell samples also showed higher expression of another cancer stem cell gene, *BMP7*, compared to CD133−/EpCAM+ cell samples (*p* = 0.0081). *OCT4* gene expression was below detection limit in all cell samples, except 5 samples (2 CD133+, 3 CD133−/EpCAM+; all from different patient tumour biopsies).

### DNA alterations in CD133+ and CD133−/EpCAM+ cell populations

The number of DNA alterations in the two cell fractions, CD133+ and CD133−/EpCAM+, displayed great heterogeneity; in the CD133+ cell population DNA alterations in the 20 patients ranged from 6 to 230 per patient (amplifications 3–18, deletions 3–212), while a range of 4–278 DNA alterations per patient (amplifications 2–17, deletions 2–261) were seen in the CD133−/EpCAM+ cell population.

**Fig. 3:**
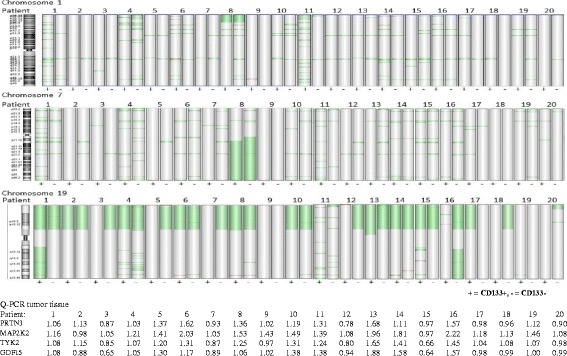
Altered chromosomes in CD133+ cells vs CD133−/EpCAM+. Chromosomal view of chromosomes with significant differences in number of alterations between CD133+ and CD133−/EpCAM+ cell fractions identified with CGH array analyses. (Green = deletions, red = amplifications). Q-PCR assay results displayed deletions and amplifications detected in tumour tissue. (Below 0.90 was regarded as deletions and above 1.10 as amplifications in Q-PCR assays)

Overall array CGH results indicated that deletions corresponded to 87% of DNA alterations in all samples; thus more common than amplifications. The total number of significant alterations (2285) in all samples was equally distributed between the two cell populations; 51% was from CD133+ population and 49% from the CD133−/EpCAM+ population (Table 2). Deletions detected in both CD133+ and CD133−/EpCAM+ [(shared deletions) and found in more than 50% (10 patients) of evaluated patients], were located on chromosome 1, 2, 7, 8, 10, 12, 14, 15, 16, 18, and 19. Amplifications detected in both CD133+ and CD133−/EpCAM+ cells [(shared amplifications) and found in more than 10 patients] were located on chromosome 3 and 14 (both related to deletions in the Agilent Euro male reference DNA) (Table 3). A list of shared deletions is supplied as Additional file 2: Table S2. Deletion of chromosome 19p occurred in 27 samples (10 CD133+ and 17 CD133−/EpCAM+) representing 18 patients (Fig. 3). A complete gene list of chromosome 19p deletion is added as Additional file 3: Table S3.

**Table 2:** Mean number of significantly altered base-pairs either specific or total (specific + shared) for CD133+ and CD133−/EpCAM+ cell fractions isolated from intraoperative colon cancer biopsies

	Specific alterations			Total alterations		
(N^°^ arrays)	CNV CD133 (20)	CNV EpCAM (20)	T-test *p* value	CNV CD133 (20)	CNV EpCAM (20)	T-test *p* value
CNV (Mbp/patient)	28.5 ± 13.6	24.8 ± 5.1	0.801	71.7 ± 22.2	68.6 ± 22.0	0.922
% of all	53	47		51	49	
Amp (Mbp/patient)	12.3 ± 11.2	2.5 ± 1.5	0.392	14.7 ± 11.5	4.63 ± 1.52	0.391
Amp % of all	83	17		76	24	
Del (Mbp/patient)	16.2 ± 5.8	22.3 ± 5.0	0.433	57.1 ± 19.4	64.0 ± 22.2	0.815
Del % of all	42	58		47	53	

mean ± SEM, CD133 = CD133^+^, EpCAM = CD133^−^/EpCAM^+^

**Table 3:** Number of patients (total 20) with either a specific CNV for each cell fraction or a shared CNV detected in both cell fractions at the same location. Note that the shared amplifications detected at chromosome 3 and 14 represents a deletion in the reference DNA

	Amplifications			Deletions		
	CD133 (20)	EpCAM (20)	Shared (20)	CD133 (20)	EpCAM (20)	Shared (20)
Chr 1	4	2	3	11	14	17
Chr 2	2	1	1	10	6	11
Chr 3	2	2	17	9	9	8
Chr 4	1	3	1	4	8	5
Chr 5	0	0	0	9	6	7
Chr 6	2	0	0	10	9	7
Chr 7	2	0	0	10	15	14
Chr 8	1	2	8	6	8	11
Chr 9	3	1	0	10	8	5
Chr 10	0	1	0	8	10	13
Chr 11	2	1	5	11	9	10
Chr 12	2	1	3	14	11	14
Chr 13	4	2	1	7	5	4
Chr 14	0	0	20	5	10	16
Chr 15	1	1	0	8	9	15
Chr 16	0	2	1	10	11	11
Chr 17	5	10	8	7	11	7
Chr 18	0	0	0	4	5	12
Chr 19	7	5	2	6	12	11
Chr 20	2	2	1	7	7	3
Chr 21	1	2	0	4	5	10
Chr 22	1	1	0	6	7	6

CD133 = CD133+, EpCAM = CD133−/EpCAM+

### DNA alterations in CD133+ versus CD133−/EpCAM+ cell populations

**Fig. 4:**
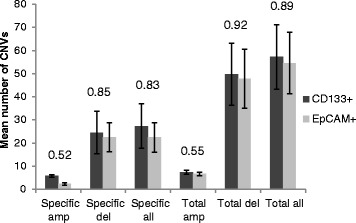
Number of DNA alterations in CD133+ versus CD133−/EpCAM+ cells. Significant differences in number of alterations were not detected between CD133+ and CD133−/EpCAM+ cell fractions. Alterations specific for either CD133+ or CD133−/EpCAM+ cell populations as well as total number of alterations (both specific and shared alterations) for each population are presented in the diagram

Amplifications were more common in CD133+ cells (83%) than in CD133−/EpCAM+ cells (17%); while deletions were more equally distributed between the cell populations, CD133+ 42% and CD133−/EpCAM+ 58% (Table 2). No significant differences in number (Fig. 4) or size (bp) of alterations between CD133+ and CD133−/EpCAM+ were detected (Table 2), except for chromosome 1, 7 and 19, which showed significantly larger alterations in CD133−/EpCAM+ population (Table 4 and Fig. 3). There were 8 alterations detected specific for CD133−/EpCAM+ cell population located at 8q24.3, 11q12.2-q13.3, 11q12.3, 11q13.4, 11q23.3, 16q13, 17p13.2 and 17q21.2. For the CD133+ cell population, 3 deletions were detected only located on 1q42.12, 8p11.22 and 12q13.13-q14.1 (Table 5).

**Table 4:** Size of CNVs (Mbp) specific for either CD133+ or CD133−/EpCAM+ cell fraction presented as amplifications and deletions per chromosome and patient

	Amplifications			Deletions		
	CD133 (Mbp/patient)	EpCAM (Mbp/patient)	T-test *p* value	CD133 (Mbp/patient)	EpCAM (Mbp/patient)	T-test *p* value
Chr 1	0.17 ± 0.1	0.06 ± 0.05	0.37	0.68 ± 0.3	1.70 ± 0.4	0.03
Chr 2	0.22 ± 0.2	0.01 ± 0.01	0.24	0.50 ± 0.3	0.24 ± 0.1	0.39
Chr 3	0.01 ± 0.008	0.009 ± 0.006	0.77	0.56 ± 0.4	0.34 ± 0.2	0.62
Chr 4	0.06 ± 0.06	0.08 ± 0.07	0.86	0.06 ± 0.03	0.04 ± 0.02	0.44
Chr 5	0	0	-	2.13 ± 1.8	0.42 ± 0.4	0.37
Chr 6	0.02 ± 0.02	0	0.17	0.21 ± 0.1	0.92 ± 0.6	0.23
Chr 7	0.01 ± 0.01	0	0.25	0.76 ± 0.4	2.23 ± 0.6	**0.05**
Chr 8	0.06 ± 0.06	0.09 ± 0.08	0.75	0.38 ± 0.2	0.16 ± 0.07	0.33
Chr 9	0.03 ± 0.02	0.01 ± 0.01	0.35	0.70 ± 0.5	1.00 ± 0.6	0.68
Chr 10	0	0.12 ± 0.12	0.32	0.37 ± 0.2	1.99 ± 1.9	0.40
Chr 11	0.02 ± 0.02	0.002 ± 0.002	0.22	1.17 ± 0.6	1.09 ± 0.5	0.91
Chr 12	6.47 ± 6.4	0.002 ± 0.002	0.32	1.06 ± 0.4	2.15 ± 9	0.27
Chr 13	4.81 ± 4.77	0.01 ± 0.008	0.32	0.08 ± 0.04	0.06 ± 0.04	0.70
Chr 14	0	0	-	0.22 ± 0.2	0.37 ± 0.2	0.53
Chr 15	0.01 ± 0.01	0.02 ± 0.02	0.80	0.45 ± 0.2	0.16 ± 0.1	0.21
Chr 16	0	0.04 ± 0.03	0.28	1.45 ± 0.6	0.49 ± 0.2	0.13
Chr 17	0.16 ± 0.09	0.23 ± 0.08	0.60	3.24 ± 2.2	0.78 ± 0.4	0.28
Chr 18	0	0	-	0.06 ± 0.04	0.13 ± 0.06	0.31
Chr 19	0.15 ± 0.06	0.08 ± 0.05	0.35	1.54 ± 1.4	7.49 ± 2.7	**0.05**
Chr 20	0.04 ± 0.03	1.54 ± 1.54	0.33	0.12 ± 0.06	0.23 ± 0.1	0.48
Chr 21	0.01 ± 0.01	0.19 ± 0.13	0.18	0.03 ± 0.03	0.002 ± 0.001	0.28
Chr 22	0.01 ± 0.01	0.006 ± 0.006	0.70	0.42 ± 0.3	0.26 ± 0.1	0.68
Total	12.3 ± 11.2	2.5 ± 1.5	0.39	16.2 ± 5.8	22.3 ± 5.0	0.43

Mean ± SEM, Mbp = mega base pairs, CD133 = CD133+, EpCAM = CD133−/EpCAM+

**Table 5:** Statistically significant DNA alterations specific for either CD133+ or CD133−/EpCAM+ cell fractions from human colon biopsies. Number of patients refers to the number in each group (*n* = 20) who displayed the alteration

Chr location	CNV	N^o^ patients CD133 (20)	N^o^ patients EpCAM (20)	N^o^ genes	Examples of genes in the region
CD133					
1q42.12	Del	7	7	7	LEFTY1/2, PYCR2, ACBD3
8p11.22	Del	5	5	2	ADAM5P, ADAM3A
12q13.13 - q14.1	Del	15	8	195	HOTAIR, GDF11, ERBB3, CDK2, CD63, PTGES3, MIR1228
EpCAM					
8q24.3	Del	7	8	27	NAPRT, MAPK15, SCRIB, PUF60, MIR661, SHARPIN
11q12.2 - q13.3	Del	9	9	247	SYT7, MIR1908, MIR612, MIR192, MIR194–2, VEGFB, MALAT1, BAD, FOSL1, RELA
11q12.3	Amp	7	5	4	SLC22A9/10/24/25
11q13.4	Del	6	8	5	PDE2A, MIR139, ARAP1, STARD10, ATG16L2
11q23.3	Del	9	8	12	BCL9L, UPK2, FOXR1, TRAPPC4
17p13.2	Del	5	8	12	PFN1, INCA1, KIF1C
17q21.2	Amp	6	11	39	Keratins and keratin-associated proteins

CD133 = CD133+, EpCAM = CD133−/EpCAM+

### DNA alterations in tumour tissue

DNA alterations in tumour tissue were detected with Q-PCR of selected by known genomic regions and on chromosome 19 in CRC. Amplifications were detected in chromosome 13 and 20 in 55% (chr13 *n* = 11, chr20 *n* = 11) of the samples (*n* = 20). Two deletions were detected in chromosome 20 and five deletions in chromosome 13. In chromosome 19p, a deletion in the specific region was detected in 6 of 20 patients (total 11 deletions in 80 reactions; 4 Q-PCR assays × 20 samples). Several tumour samples displayed amplification in tumour tissue at chromosome 19p (50%, *n* = 40 of 80 reactions, representing 10 of 20 patients) (Fig. 2).

## Discussion

Colorectal cancer is genetically recognised with many reported alterations and mutations. However, most genomic analyses have been performed on tumour tissue material – with thousands or millions of cells analysed mixed together. Tumour tissue consists of several different cell types beside tumour cells, which may also be heterogeneous. Therefore, our aim was to compare genomic alterations in two well-defined cell fractions consisting of CD133+ and CD133−/EpCAM+ enriched cells.

CD133, also known as prominin-1, is considered to be a cancer stem cell marker in fresh, surgically resected colorectal cancer samples [3]. It is widely used to identify and isolate cancer stem cells with prediction of survival, recurrence, metastasis and chemotherapy resistance [11]. Also, in tumour tissue, CD133 is localized in areas with high cellularity while it is hardly detected at all in mucosa [4]. In the present paper, other stem cell markers such as BMP7 was also used to confirm that CD133+ cells are more stem cell like than CD133−/EpCAM+ cells. BMP7 is a regulator involved in the maintenance of the stem cell niche and related to poor prognosis in colorectal cancer patients [12]. OCT4 is regarded a key factor in maintenance of pluripotency of stem cells and has been related to poor prognosis in cancer [13, 14]. There was a significant difference in expression of *BMP7* between the two cell populations with high expression in the CD133+ fraction, while *OCT4* was hardly observed in any of the cell populations.

Reported studies on colorectal cancer tissue display amplifications at chromosome 8q, 13 and 20q, and deletions at chromosome 8p, 17p and 18q [15–17]. Amplifications at chromosome 13 and 20 were detected in 11 of our 20 patients at tumour tissue analyses while such alterations were not detected to the same extent in the two cell fractions; amplifications at chromosome 13 were detected in 6 patients, and 4 patients had amplifications at chromosome 20. In this study, we detected a deletion at chromosome 19p in 17 of 20 patients in both CD133+ and CD133−/EpCAM+ cells. This deletion was only detected in 6 of 20 patients at tumour tissue level with Q-PCR, while 10 patients displayed a gain instead (Fig. 2). In our earlier studies on genomic alterations in colorectal cancer we detected gain at chromosome 19p13.3 in both tumour tissue and adjacent mucosa, which was more common in advanced cancer (Dukes D patients) [15]. A deletion at chromosome 19 in colorectal cancer has only been reported in a study by Cimino Reale et al. in 3 of 11 patients [18], while a gain of 19p was detected in both primary and recurrent tumour tissue by others [19]. It is possible that the deletion may be diluted by a bulk of other cells in tumour tissue and thereby escaped detection, especially considering that a gain at 19p was earlier detected by ourselves in the mucosa of colorectal cancer patients [15]. Chromosome 19 is one of the most gene dense chromosomes and a deletion of the p-arm affects several genes (Additional file 3: Table S3). Chromosomal fragile sites are unstable genomic regions prone to gaps or breaks. In cancer, these regions are frequently sites of chromosomal rearrangements including translocations, deletions, and amplifications [20]. On chromosome 19, fragile sites have been found at 19p13.1 and q13 as well as in the centromeric region, 19p11/q11.

Overall, deletions were more common than amplifications in our CD133 positive and negative cell fractions opposing our earlier result on mixed tumour tissue [15]. An explanation may be that DNA deletions were diluted by the presence of normal DNA. Otherwise DNA alterations were in number and size distributed equally between the two cell fractions. However, large alterations were detected at chromosome 1, 7 and 19 in CD133−/EpCAM+ cell population. Also, several large amplifications were detected in CD133+ cells compared to CD133−/EpCAM+ cells, although not significantly different probably due to high genetic heterogeneity displayed in both cell fractions. Some patients had numerous alterations while others had few; a span of more than 200 alterations was found between patients with few versus high frequency of DNA alterations. An explanation for this could be chromosomal instability that is common in colorectal cancer [21].

Our present aim was to compare differences in genomic alterations between CD133+ and CD133−/EpCAM+ cell fractions. Therefore, we focused on specific alterations in the two cell fractions. However, some patients had similar genomic alterations in the two cell fractions while others had completely different alterations. This made it hard to say that CD133+ cells are genetically homogenous or defined as stated by Gaiser et al. who concluded aberrant profiles between CD133+ and CD133- cells in 7 out of 12 patient samples [22]. Due to the amount of alterations detected among our patients it would require very large groups of patients to confirm whether CD133+ cells are genetically distinct. Great genetic heterogeneity may represent different subclones of CD133+ cells and CD133−/EpCAM+ cells [23].

In this study, specific alterations detected for CD133+ were deletions of 1q, 8p and 12q while specific alterations for CD133−/EpCAM+ cell fractions were deletions of 8q, 11q and 17p, and amplifications on 11q and 17q. Deleted DNA regions in CD133+ cell population contain known cancer related factors such as ERBB3 (HER3) and HOTAIR [24, 25]. In CD133−/EpCAM+ cells significantly altered DNA regions contained factors known to be involved in cancer such as VEGFB and MIR192 [26, 27] (Table 4). However, these alterations occurred in both cell fractions from patients at individual analyses of the arrays. Therefore, a most relevant change for tumour progression may be the deletion at chromosome 19p that was detected in both cell fractions in 90% of our patients involving 575 genes and miRNAs.

## Conclusion

Separation of solid tumour tissue into defined enriched cell fractions displayed genomic alterations that were not observed in mixed tumour tissue. In cell fractions of CD133+ and CD133−/EpCAM+ cells we detected a deletion at chromosome 19p that was not evident in corresponding tumour tissue. Importantly, CD133+ cells did not show a distinct genetic profile, but displayed several alterations (mostly deletions) with large heterogeneity both within tumours from the same patient and among patients.

## Additional files

Additional file 1: Table S1. Primer sequences for chromosome 19p Q-PCR on tumor tissue. (DOCX 19 kb)

Additional file 2: Table S2. Shared deletions among CD133+ and CD133−/EpCAM+ cell fractions analyzed with inter-array analysis. Chromosomal location and size (bp) of shared deletions. (DOCX 20 kb)

Additional file 3: Table S3. Chromosome 19p gene list (575 genes in the deleted region at chromosome 19p). Name of the genes located in the deleted region at chromosome 19p. (DOCX 27 kb)

## Abbreviations

aCGH: Array comparative genome hybridization
ALP: Alkaline phosphatase
CNV: Copy number variation
CSC: Cancer stem cells
EpCAM: Epithelial cell adhesion molecule
ICC: Immunocytochemistry
LPA: Linear polyacrylamide
PROM1: Prominin 1
