# Supplementary material for: DNA alterations in Cd133+ and Cd133- tumour cells enriched from intra-operative human colon tumour biopsies
Source: BMC Cancer. 2017 Mar 27;17:219. doi: 10.1186/s12885-017-3206-8 (PMC5369016; doi:10.1186/s12885-017-3206-8)
Supplement: Supplementary file 1 — Primer sequences for chromosome 19p Q-PCR on tumor tissue. (DOCX 19 kb) [file 12885_2017_3206_MOESM1_ESM.docx]

Table S1. Primer sequences for chromosome 19p Q-PCR on tumor tissue.

| Assay | Primer | Sequence (5´→3´) |
| --- | --- | --- |
| Ch19PRTN3 | Ch19PRTN3_fw  Ch19PRTN3_rv | CCT GGT GAA CGT GGT GCT  TCT CCG CGT CGT AGT TGT TC |
| Ch19MAP2K2 | Ch19MAP2K2_fw  Ch19MAP2K2_rv | TTG AGG GGT TCA TGG TGT AGA AG  CTG GAT TCT GGT CAT TGG TGT GT |
| Ch19TYK2 | Ch19TYK2_fw  Ch19TYK2_rv | CTT TCG CGG AGC CTA CTT CC  TGC AAA CTT CAC TCC GAC CC |
| Ch19GDF15 | Ch19GDF15_fw  Ch19GDF15_rv | CCT GCA CCT GCG TAT CTC TC  TGT CAC GTC CCA CGA CCT T |
