# Supplementary material for: DNA alterations in Cd133+ and Cd133- tumour cells enriched from intra-operative human colon tumour biopsies
Source: BMC Cancer. 2017 Mar 27;17:219. doi: 10.1186/s12885-017-3206-8 (PMC5369016; doi:10.1186/s12885-017-3206-8)
Supplement: Supplementary file 2 — Shared deletions among CD133+ and CD133−/EpCAM+ cell fractions analyzed with inter-array analysis. Chromosomal location and size (bp) of shared deletions. (DOCX 20 kb) [file 12885_2017_3206_MOESM2_ESM.docx]

Table S2. Shared deletions among CD133+ and CD133-/EpCAM+ cell fractions analyzed with inter-array analysis.

| Location | Size (bp) |
| --- | --- |
| 1q21.3-23 | 3657007 |
| 2q22.3 | 59156 |
| 2q35 | 746362 |
| 3p25.3 | 202518 |
| 6q23.3 | 17502 |
| 7p15.2 | 182344 |
| 7q22.1 | 2506700 |
| 8p23.1 | 12760 |
| 10q22.3 | 122200 |
| 11q13.1 | 226788 |
| 12p13.31 | 76102 |
| 15q22.31 | 89381 |
| 15q26.2 | 45329 |
| 16p13.3 | 73458 |
| 16p11.2 | 2970287 |
| 16q23.2 | 32739 |
| 18q12.2 | 298442 |
| 19p13.3-13.11 | 19133209 |
| 21p11.2 | 2234 |
| 22q13.2 | 34472 |
