# Supplementary material for: DNA alterations in Cd133+ and Cd133- tumour cells enriched from intra-operative human colon tumour biopsies
Source: BMC Cancer. 2017 Mar 27;17:219. doi: 10.1186/s12885-017-3206-8 (PMC5369016; doi:10.1186/s12885-017-3206-8)
Supplement: Supplementary file 3 — Chromosome 19p gene list (575 genes in the deleted region at chromosome 19p). Name of the genes located in the deleted region at chromosome 19p. (DOCX 27 kb) [file 12885_2017_3206_MOESM3_ESM.docx]

Table S3. Chromosome 19p gene list (575 genes in the deleted region at chromosome 19p).

| MEF2B | SH3GL1 | OR7E24 | FEM1A | PGLYRP2 | KRI1 | STXBP2 |
| --- | --- | --- | --- | --- | --- | --- |
| LSM7 | ZNF77 | RDH8 | ZNF653 | RLN3 | ANGPTL4 | SH2D3A |
| ZNF57 | CRTC1 | KISS1R | CCL25 | PDE4A | TMEM59L | ATG4D |
| USE1 | RAD23A | CILP2 | WDR18 | CDKN2D | MIR1181 | HDGF2 |
| FSTL3 | CCDC94 | ZNF878 | MPV17L2 | ZNF442 | GLT25D1 | SLC35E1 |
| GRIN3B | ACSBG2 | TICAM1 | REXO1 | ZNF443 | RAB8A | HAUS8 |
| SNORA68 | ACP5 | ZNF491 | RAB11B | CIB3 | OR1I1 | NCAN |
| ANKLE1 | SNORD41 | FBXL12 | ILVBL | INSR | FLJ25758 | CARM1 |
| TMEM146 | XAB2 | BRD4 | TRIP10 | EBI3 | AKAP8L | WIZ |
| HMHA1 | C19orf22 | DPP9 | ZNF561 | MATK | ARID3A | FKBP8 |
| PGPEP1 | EMR4P | ZNF490 | ZNF562 | TMEM205 | SPPL2B | AP1M1 |
| DIRAS1 | C19orf23 | MIR639 | PCSK4 | BST2 | CRB3 | AP1M2 |
| C19orf44 | C19orf24 | MIR637 | ZNF266 | ZNF441 | MYO1F | PRSSL1 |
| C19orf45 | C19orf25 | MIR638 | ZNF560 | ZNF440 | ARMC6 | CRLF1 |
| MED26 | RPS28 | KIAA0892 | ZNF564 | STAP2 | VAV1 | MIR7-3 |
| BTBD2 | C19orf26 | MRI1 | ZNF563 | EEF2 | DOCK6 | LOC100131801 |
| LSM4 | LYL1 | MRPL4 | HAPLN4 | PDE4C | SAFB2 | TIMM13 |
| C19orf42 | C19orf28 | ARHGEF18 | UPF1 | RNASEH2A | DNASE2 | RNF126 |
| C19orf43 | ZNF799 | ELANE | PPAN-P2RY11 | TECR | DOHH | CCDC124 |
| MAP2K7 | C19orf29 | TLE6 | TMPRSS9 | LOC100130932 | ZNF317 | LRG1 |
| LPHN1 | SFRS14 | FLJ25328 | ELAVL1 | PNPLA6 | GTF2F1 | ADAT3 |
| GNG7 | TCF3 | NDUFA13 | ELAVL3 | JUNB | TBXA2R | TMED1 |
| NWD1 | KIAA1683 | SNORD37 | SF3A2 | DDX39 | DNAJB1 | ADAMTS10 |
| ZNF44 | C19orf21 | TLE2 | KANK3 | ZNF439 | ZNF414 | FBN3 |
| PTBP1 | ZNF69 | ZNF333 | ZNF20 | ZNF433 | CACNA1A | LOC55908 |
| USHBP1 | FAM108A1 | ZFR2 | ZNF358 | CALR3 | IER2 | ALKBH7 |
| PTPRS | ZNF625 | ZSWIM4 | SGTA | SNORD105 | LRRC8E | OR7A5 |
| ZNF791 | PLEKHJ1 | LRRC25 | FSD1 | UBA52 | PODNL1 | TSPAN16 |
| HMG20B | **MAP2K2** | NDUFA11 | DOT1L | SMARCA4 | PIP5K1C | TNFSF9 |
| MIR220B | ZNF121 | MAST3 | 02-mar | ABCA7 | PRDX2 | COL5A3 |
| ZNF788 | ACER1 | C19orf10 | LOC729991-MEF2B | STK11 | HSD11B1L | ACTL9 |
| RETN | KLF16 | MAST1 | P2RY11 | LOC113230 | PEX11G | BRUNOL5 |
| CCDC130 | ZNF627 | ZNF136 | GAMT | ADAMTSL5 | NDUFS7 | OR1M1 |
| C19orf59 | CCDC159 | ZNF555 | ZNF763 | TMIGD2 | CCDC105 | KDM4B |
| C19orf57 | C19orf6 | MIDN | NCLN | MIR181C | SLC1A6 | EPOR |
| CD320 | C19orf35 | ZNF554 | KIAA1543 | ZNF699 | PCP2 | JAK3 |
| MED16 | C19orf36 | ABHD8 | DNM2 | FBXW9 | DDA1 | **GDF15** |
| RPS15 | C19orf34 | APC2 | GNA15 | HNRNPM | MAN2B1 | GDF1 |
| ZGLP1 | C19orf39 | ZNF559 | CHERP | ANGPTL6 | LOC388499 | EFNA2 |
| C19orf56 | CD209 | C3 | SLC44A2 | ISYNA1 | ANO8 | THOP1 |
| C19orf53 | C19orf38 | ZNF558 | GNA11 | IL12RB1 | DCAF15 | NR2C2AP |
| TRAPPC5 | CCDC151 | ZNF557 | RGL3 | OAZ1 | SSBP4 | ZNRF4 |
| FDX1L | UCA1 | ZNF556 | TNFSF14 | DDX49 | LOC126536 | LPAR2 |
| C19orf52 | C19orf30 | MKNK2 | PRKCSH | MIR181D | PTGER1 | S1PR2 |
| C19orf50 | SNORD105B | RPL36 | MOBKL2A | JUND | LOC729991 | FAM125A |
| TM6SF2 | LDLR | QTRT1 | KANK2 | ZNF426 | TMEM38A | ATCAY |
| UNC13A | OCEL1 | ZNF177 | UQCR11 | ASF1B | PKN1 | HOOK2 |

Continues on next page

| RTBDN | PLK5P | AZU1 | OR2Z1 | KHSRP | KCNN1 |
| --- | --- | --- | --- | --- | --- |
| SIN3B | DENND1C | BEST2 | ZNF844 | DHPS | MAP1S |
| SHD | NOTCH3 | PLIN5 | FZR1 | CLEC4GP1 | SYDE1 |
| HOMER3 | SEMA6B | PLIN4 | TNFAIP8L1 | COPE | CASP14 |
| S1PR4 | ATP13A1 | PLIN3 | ONECUT3 | NANOS3 | CYP4F3 |
| CLEC4G | RPL18A | MIR1909 | GIPC3 | GPR108 | CYP4F2 |
| RAVER1 | KLF2 | GATAD2A | GIPC1 | SBNO2 | EVI5L |
| S1PR5 | CHAF1A | FUT3 | MUM1 | NDUFB7 | MPND |
| OR7C1 | KLF1 | APBA3 | KEAP1 | C19orf70 | NFIC |
| OR7C2 | ATP5D | MLLT1 | DAZAP1 | C19orf71 | TSSK6 |
| CREB3L3 | SLC27A1 | AP3D1 | ZNF846 | PLVAP | MUC16 |
| CFD | IL27RA | CC2D1A | SPC24 | NFIX |  |
| MRPL34 | TMEM161A | MIR24-2 | GADD45GIP1 | C19orf77 |  |
| MORG1 | TRMT1 | FAM32A | GMIP | TPM4 |  |
| GTPBP3 | RFXANK | MIR199A1 | EIF3G | RAX2 |  |
| CLEC4M | LONP1 | NXNL1 | RASAL3 | LINGO3 |  |
| PGLS | AES | CYP4F11 | B3GNT3 | LOC147727 |  |
| UHRF1 | OR7G3 | ILF3 | REEP6 | ZNF709 |  |
| PIAS4 | ELOF1 | CYP4F12 | DAND5 | COMP |  |
| PRAM1 | GPX4 | SAMD1 | ZNF833 | SAFB |  |
| OR7D2 | NR2F6 | YJEFN3 | EPHX3 | PALM3 |  |
| OR7D4 | FAM129C | MIR1470 | SYCE2 | CNN2 |  |
| LPPR3 | TUBB4 | MEX3D | EMR1 | CNN1 |  |
| C3P1 | ICAM1 | UBXN6 | LASS4 | SLC25A42 |  |
| PALM | SNAPC2 | TJP3 | LASS1 | TNPO2 |  |
| LPPR2 | ICAM4 | LOC100128573 | RFX1 | SLC39A3 |  |
| ELL | ICAM5 | PBX4 | CIRBP | ZNF700 |  |
| MIR1227 | ICAM3 | F2RL3 | DNMT1 | PIK3R2 |  |
| LMNB2 | ARRDC5 | OR7A17 | YIPF2 | SLC25A41 |  |
| FCER2 | MBD3 | NRTN | EMR3 | DUS3L |  |
| IFI30 | ECSIT | ZNF823 | EMR2 | RPL18AP3 |  |
| MYO9B | ARRDC2 | JSRP1 | RFX2 | PLAC2 |  |
| MIR27A | ANKRD24 | SLC25A23 | ASNA1 | ZBTB7A |  |
| CD70 | FARSA | PRKACA | INSL3 | NACC1 |  |
| CALR | GADD45B | MCOLN1 | SLC5A5 | C19orf62 |  |
| CDC37 | CAPS | SCAMP4 | EPS15L1 | C19orf60 |  |
| PIN1 | RAB3A | OR7A10 | CD97 | NDUFA7 |  |
| PPAN | POLR2E | MBD3L1 | CLEC17A | OR10H1 |  |
| MRPL54 | RAB3D | VMAC | KLHL26 | SIRT6 |  |
| STX10 | **PRTN3** | MBD3L3 | RANBP3 | OR10H5 |  |
| OLFM2 | FUT6 | MBD3L2 | ATP8B3 | OR10H4 |  |
| FCHO1 | FUT5 | MBD3L5 | DAPK3 | OR10H3 |  |
| PRR22 | CYP4F22 | CSNK1G2 | TIMM44 | OR10H2 |  |
| GCDH | CTXN1 | MBD3L4 | **TYK2** | UBL5 |  |
| OR7G2 | HSH2D | FGF22 | AMH | C19orf66 |  |
| OR7G1 | MIR23A | CLPP | ITGB1BP3 | CYP4F8 |  |
| MIR1238 | SF4 | AKAP8 | CPAMD8 | PSPN |  |

**Primers for Q-PCR assays**
